# Antifungal activity of flavonoids and modulation of expression of genes of fatty acid synthesis in the dermatophyte *Trichophyton rubrum*

**DOI:** 10.1186/1753-6561-8-S4-P53

**Published:** 2014-10-01

**Authors:** Tamires Aparecida Bitencourt, Tatiana TakahasiKomoto, Mozart Marins, Ana Lúcia Fachin

**Affiliations:** 1 University of Ribeirão Preto, Ribeirão Preto, São Paulo,14096900, Brazil

## Background

Dermatophytosis are fungal infections caused by keratinophilic fungi known as dermatophytes and classified in three genera: *Trichophyton, Epidermophyton and Microsporum. Trichophyton rubrum *is the most frequent species associated to dermatophytosis worldwide [1]. The infections caused by dermatophytes are not lethal, but are difficult to treat and uncomfortable. In the case of *T. rubrum*, they tend to be chronic, and although the superficial infections are more common, cases of deep infection have been reported in immunocompromised patients [2][3]. The number of antifungal drugs are still limited, and the acquired resistance for some of clinical antifungal have been shown as well as the side effects that have been promoted by them. Reasons for the challenge in development of new antifungal drugs are the similarities shared by fungal and mammalian cells and the lack of knowledge about the biology of these pathogens. Recent evidences have shown that the fatty acid sinthase (FAS) is an interesting antifungal target [4] because of marked differences between human and fungal cells. The aim of this study was to evaluate the antifungal activity of four flavonoids described as FAS inhibitors and verify the modulation of genes in the pathway of fatty acid synthesis in *T. rubrum *growth in presence of the most effective one as FAS inhibitor.

## Methods

The susceptibility assay was carried out using the microdilution test in RPMI medium in 96-well plates with the flavonoids quercetin, ellagic acid, galangin and genistein in a range of 1000-1.9µg/mL toward the strain ATCC MYA-3108 of *T. rubrum *[5]. The modulation of genes fatty acid synthesis(FAS 1p, FAS 2p subunits), acetyl-COA carboxylase 2p subunity and fatty acid transporter protein (*FAT*1) was analyzed by quantitative PCR using Sybr Green after 16h of incubation of strain of *T.rubrum *with MIC of quercetin in liquid Sabouraud medium.

## Results and conclusion

Quercetin showed the most effective antifungal activity with MIC of 125 µg/mL, ellagic acid presented MIC of 250 µg/mL, galangin and genistein were ineffective toward *T.rubrum (*MIC >1000 µg/mL). The positive controls fluconazole and cerulenin presented MICs of 63 and 125 µg/mL, respectively. The analyse of gene expression of the fatty acid synthetic pathway showed the majority of the genes were downregulated by quercetin, fluconazole and cerulenin. However, cerulenin caused a low upregulation of *FAS*2p gene. Thus, the results suggest the activity of quercetin could be due the modulation of genes of the pathway of fatty acid synthesis, which is a fungal specific target for development of antifungal drugs.
